# Prevalence of multidrug-resistant tuberculosis among Category II pulmonary tuberculosis patients

**Published:** 2011-03

**Authors:** Surendra K. Sharma, Sanjeev Kumar, P.K. Saha, Ninoo George, S.K. Arora, Deepak Gupta, Urvashi Singh, M. Hanif, R.P. Vashisht

**Affiliations:** *Department of Medicine, All India Institute of Medical Sciences, New Delhi, India*; * *Department of Microbiology, All India Institute of Medical Sciences, New Delhi, India*; ** *Sanjay Gandhi Memorial Hospital, New Delhi, India*; † *New Delhi Tuberculosis Centre, New Delhi, India*

**Keywords:** Category II patients, India, multidrug-resistant tuberculosis, previously treated TB patients, pulmonary tuberculosis

## Abstract

**Background & objectives:**

Multidrug-resistant tuberculosis (MDR-TB) has emerged as a significant global health concern. The most important risk factor for the development of MDR-TB is previous anti-tuberculosis therapy. Category II pulmonary TB includes those patients who had failed previous TB treatment, relapsed after treatment, or defaulted during previous treatment. We carried out this study to ascertain the prevalence of MDR-TB among category II pulmonary TB patients.

**Methods:**

This was a cross-sectional, descriptive study involving category II pulmonary TB patients diagnosed between 2005 and 2008. All sputum-positive category II TB cases were subjected to mycobacterial culture and drug-susceptibility testing (DST). MDR-TB was defined as TB caused by bacilli showing resistance to at least isoniazid and rifampicin.

**Results:**

A total of 196 cases of sputum-positive category II pulmonary tuberculosis patients were included. Of these, 40 patients (20.4%) had MDR-TB. The mean age of MDR-TB patients was 33.25 ± 12.04 yr; 9 patients (22.5%) were female. Thirty six patients showed resistance to rifampicin and isoniazid; while 4 patients showed resistance to rifampicin, isoniazid and streptomycin. The prevalence of MDR-TB among category-II pulmonary tuberculosis patients was 20.4 per cent.

**Interpretation & conclusions:**

The prevalence of MDR-TB in category II TB patients was significant. However, nation-wide and State-wide representative data on prevalence of MDR-TB are lacking. We stress the importance of continuous monitoring of drug resistance trends, in order to assess the efficacy of current interventions and their impact on the TB epidemic.

Multidrug-resistant tuberculosis (MDR-TB) has emerged as a significant global health concern^1^,^2^. There are alarming reports of increasing drug resistance from various parts of the globe which potentially threaten to disrupt the gains achieved in tuberculosis (TB) control over the last decade^3^. MDR-TB is essentially a man-made phenomenon and arises due to inadequate treatment of drug-sensitive TB^4^. The prevalence of MDR-TB mirrors the functional state and efficacy oftuberculosis control programmes in the country. Previous treatment for TB is the strongest risk factor for development of MDR-TB^5^. Category II pulmonary TB includes those patients who had failed previous TB treatment, relapsed after treatment, or defaulted during previous treatment^6^. Since such patients have already been exposed to anti-tuberculosis agents, they are at high risk for harbouring multi-drug resistant strains. Therefore, it is imperative to know the prevalence of MDR-TB among category II pulmonary TB patients. The present study focuses on the prevalence of MDR-TB and pattern of drug resistance among category II pulmonary TB patients from a tertiary care centre and a primary care level centre in northern India.

## Material & Methods

This cross-sectional, descriptive study involved category II sputum positive pulmonary tuberculosis patients, aged 18 to 60 yr. Standard definitions for treatment failure, relapse and default were used^6^. The cases were recruited between March 2005 and March 2008 through the out-patient department of All India Institute of Medical Sciences (AIIMS) hospital, New Delhi, and a dedicated chest clinic functioning at primary care level at Sanjay Gandhi Memorial Hospital in Mangolpuri, New Delhi. Data of this report were derived from an ongoing trial that is being done to see the effect of *Mycobacterium w* vaccination in category II pulmonary TB patients.

The following patients were excluded from this study: (*i*) presence of secondary immunodeficiency states like HIV, organ transplantation, diabetes mellitus, malignancy, treatment with cytotoxic drugs currently or within last 3 months, use of corticosteroids; (*ii*) hepatitis B or C co-infection; (*iii*) alcoholism; (*iv*) extra-pulmonary TB and/or patients requiring surgical intervention; (*v*) seriously ill and moribund patients with very low lung reserve and BMI < 15 kg/m ^2^ (initially patients were recruited with BMI <15); (*vi*) pregnancy and lactation; (vii) known seizure disorder; (*viii*) known symptomatic cardiac disease, such as arrhythmias or coronary artery disease; (ix) abnormal renal function (serum creatinine >2 mg/dl; >2+ proteinuria); (x) abnormal hepatic function (bilirubin > 1.5 mg/dl; AST, ALT, SAP more than 1.5 × ULN; PT = 1.3 × control); (xi) Patients with haematological abnormalities (WBC less than or equal to 3000/mm ^3^ ; platelets less than or equal to 100,000/mm^3^).

The study protocol was approved by the ethics committee of the institute. A written informed consent was taken from each patient for inclusion in the study. All patients were subjected to sputum-smear microscopy for acid-fast bacillus (AFB) and chest radiography at the time of enrollment in category II treatment for the study. All sputum specimens were subjected to culture on Lowenstein-Jensen (L-J) slopes by Petroff’s method. n0 iacin test, catalase test and para-nitrobenzoic acid (PNB) test were used to identify the isolated mycobacteria. The positive cultures were evaluated for drug susceptibility pattern at New Delhi Tuberculosis Center laboratory, New Delhi which is an accredited intermediate reference laboratory (IRL) for mycobacterial culture and drug susceptibility testing (DST). DST was carried out by economic variant of 1 per cent proportion method. The sensitivity tests were set up with inoculum prepared from the growth of selected positive slopes. The standard reference strain H37Rv was tested in addition with each batch of tests. The inoculated slopes were evaluated for growth after 28 and 42 days of incubation. DST was also carried out at AIIMS hospital, New Delhi. However, for this communication we have used data from New Delhi Tuberculosis Center laboratory, New Delhi as AIIMS hospital laboratory was under accreditation process.

## Results

A total of 445 category II pulmonary TB patients were screened between 2005 and 2008; 249 patients were excluded due to various reasons (Fig.). Finally, 196 category II sputum positive pulmonary TB patients were included in the study. Their baseline characteristics are shown in Table I. MDR-TB was detected in 40 (20.4%) patients. The mean age of MDR-TB patients was 33.25 ± 12.04 (18-55) yr with BMI of 17.84 ± 2.4 kg/m^2^. Of these 40 patients, 29 (72.5%) had relapse, 3 (7.5 %) had treatment failure and 8 patients (20%) were defaulters. Nine patients (22.5%) were female. Thirty six patients showed resistance to rifampicin and isoniazid; 4 patients showed resistance to streptomycin (in addition to rifampicin, isoniazid). Thus, the prevalence of MDR-TB among category-II pulmonary tuberculosis patients was 20.4 per cent. The pattern of anti-tuberculosis drug resistance among category II pulmonary TB patients is shown in Table II. Prevalence of MDR-TB in various subcategories of category-II pulmonary TB patients is shown in Table III.

**Fig. 1:**
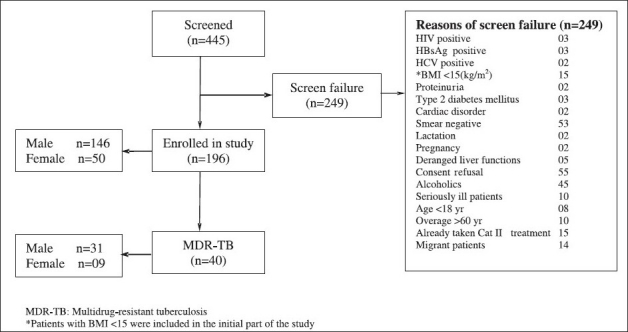
Flow chart showing detailed break-up of patients.

**Table I:** Baseline characteristics of 196 category II pulmonary TB patients

Baseline characteristic	Category II pulmonary TB patients

Age (yr)	31.97 ± 10.3 (18−58)*
Sex	
Male (%)	146 (74.5)
Female (%)	50 (25.5)
Body mass index (kg/m^2^)	18.8 ± 3.9 (13.45 − 26.90*
Relapse (%)	147 (75)
Treatment after default (%)	33 (16.8)
Treatment failure (%)	16 (8.2)

* mean ± SD (range)

**Table II:** Pattern of drug resistance among category II pulmonary TB patients

Pattern of drug resistance	Cat II patients No. (%)

RH	36 (18.4)
RHS	04 (2.04)
R	03 (1.5)
H	0
S	0

R, rifampicin; H, isoniazid; S, streptomycin

**Table III:** MDR-TB in various subcategories of Cat II pulmonary TB patients

Sub-category	Total patients	MDR−TB No. (%)

Relapse	147	29 (19.7)
Treatment after default	33	08 (24.2)
Treatment failure	16	03 (18.7)
Total	196	40 (20.4)

**Table IV:** Prevalence of MDR-TB among previously treated cases of pulmonary TB in Indias

Location	Period of study	No. of isolates	MDR-TB(%)

Gujarat^7^	1983−1986	1259	30.2
Delhi^8^	1990−1991	81	33.3
Haryana^9^	1991−1995	196	49
Tamil Nadu^10^	1996	162	20.3
Delhi^11^	1996−1998	263	14
Bangalore^12^	1999−2000	226	12.8
Tamil Nadu†	1999−2003	440	11.8
Ahmadabad^13^	2000−2001	822	37
Gujarat^3^	2002−2007	1047	17.2
Delhi^14^	2006	2880	47.1
Present study	2005−2008	196	20.4

† Tuberculosis Research Centre (TRC), Chennai, unpublished data

## Discussion

The present study showed the prevalence of MDR-TB among category II pulmonary TB patients as 20.4 per cent. This was comparable to MDR-TB rates published in previous studies from India^7^–^14^. Studies conducted over the past two decades have shown MDR-TB rates varying from 14 to 49 per cent among previously treated cases (Table IV). The World Health Organization (WHO) fourth Global Project reported a MDR-TB prevalence of 17.2 per cent among previously treated cases in India^3^.

Our findings carry significant importance because there have been scarce data on the prevalence of MDR-TB among category II pulmonary TB patients from the recent past. Since drug-resistance is a dynamic phenomenon, it is important to monitor the trend of drug-resistance periodically. Moreover, our study was a prospective study conducted over a period of three years.

Findings of the present study have to be interpreted cautiously in the light of certain limitations. First of all, this is a hospital-based study and hence there could have been significant referral bias involved in patient selection. Secondly, these results cannot be extrapolated to category II patients in other parts of the country. Thirdly, rigorous exclusion criteria were applied to screen patients, which may not be applicable in real-life situation.

In conclusion, our findings showed that the prevalence of MDR-TB in category II TB patients was high and these patients are at high risk of amplified resistance including XDR-TB^15^. A large multi-centric study involving patients recruited at primary-care level from different parts of the country is needed to determine the nation-wide prevalence of MDR-TB in category II TB patients. Findings of this report suggest that all category II PTB patients, given the high prevalence of MDR-TB, should be screened for MDR-TB using rapid diagnostic tests (molecular tests) such as the line probe assays. This will facilitate the diagnosis of MDR-TB at an early stage and thus will minimize transmission of the disease^16^. We stress the importance of continuous monitoring of drug resistance trends, in order to assess the efficacy of current interventions and their impact on the TB epidemic.
